# Medico-Legal Question

**Published:** 1848-07

**Authors:** 


*Medico-Legal Question.*—There is now pending, in the (*ci-devant*) Cour Royale of Poitiers, a case of appeal which is interesting in a medico-legal point of view. Dr. Vivielle has been fined in costs, by a lower court, for refusing to give evidence as a witness, he considering the confidence placed in him by his patient, to be of a sacred nature. He had attended a Mr. B—— for a venereal affection, and the wife having been injured in her health by this circumstance, was also obliged to put herself under his care. She now sought a separation, and demanded from her medical attendant that he should disclose to the court every circumstance connected with herself and husband, which, as above stated, Dr. Vivielle refused to do.

The Medical Society of La Rochelle, where the parties are residing, has taken up the matter, and its members have testified to their associate their approval of his conduct. We must not omit to add, that this same Cour Royale of Poitiers gave, in 1828, a verdict in favour of the secrecy of medical men, in certain circumstances. Several letters have appeared in the French medical journals, advocating different views on the question, and the celebrated Lamenais has been made to give his opinion. He very justly remarks "that a medical man has two distinct duties to perform—one towards his patient, the other towards society; and if it be incumbent upon him in the latter capacity, to apprise the authorities of the existence of a contagious disease, it is equally right and just that he should disclose any circumstance which might lead to the detection of wrong." In our own country, a medical witness who refused to give evidence when called on in a court of law, would be liable to imprisonment for contempt of court.—*Ibid.*

